# Plasma Activation of Copper Nanowires Arrays for Electrocatalytic Sensing of Nitrate in Food and Water

**DOI:** 10.3390/nano9020150

**Published:** 2019-01-25

**Authors:** Angela Maria Stortini, Sabrina Fabris, Gloria Saorin, Emanuele Verga Falzacappa, Ligia Maria Moretto, Paolo Ugo

**Affiliations:** 1 Department of Molecular Sciences and Nanosystems, University Ca’ Foscari of Venice, 30172 Venice, Italy; stortini@unive.it (A.M.S.); sabri@unive.it (S.F.); 846278@stud.unive.it (G.S.); ugo@unive.it (P.U.); 2 Nadir S.r.l. Plasma & Polymers, 30172 Venice, Italy; emanuele.verga@unive.it

**Keywords:** nitrate, copper, nanowires, template, food, low-temperature plasma

## Abstract

Electrochemical methods for nitrate detection are very attractive since they are suitable for in-field and decentralized monitoring. Copper electrodes are often used to this aim as this metal presents interesting electrocatalytic properties towards nitrate reduction. In this research, we study improvements in the electrochemical analysis of nitrate in natural water and food by taking advantage of the detection capabilities of ensembles of copper nanowire electrodes (CuWNEEs). These electrodes are prepared via template electrodeposition of copper within the nanopores of track-etched polycarbonate (PC) membranes. A critical step in the preparation of these sensors is the removal of the template. Here, we applied the combination of chemical etching with atmospheric plasma cleaning which proved suitable for improving the performance of the nanostructured copper electrode. Analytical results obtained with the CuWNEE sensor for nitrate analyses in river water samples compare satisfactorily with those achieved by standard chromatographic or spectroscopic methods. Experimental results concerning the application of the CuWNEEs for nitrate analysis in food samples are also presented and discussed, with focus on nitrate detection in leafy vegetables.

## 1. Introduction

The presence of nitrate in water and vegetables is generally associated with soil fertilization. Concerning edible vegetables, higher levels of nitrate are detected in the leaves, while it is well documented that wash-out waters from agricultural lands can be enriched with nitrate [1,2]. Toxicity of nitrate to humans is related to its ability to oxidize hemoglobin (Hb) to methemoglobin (metHb), which is unable to transport oxygen in the tissues. This process is responsible for “blue-baby” syndrome or methemoglobinemia [1,3]. Moreover, in the human body, nitrate can be converted to metabolites, such as nitrite, nitric oxide and N-nitroso compounds, which are responsible for potentially adverse health implications, such as carcinogenic effects on the gastrointestinal apparatus (nitrite as a precursor for endogenous nitrosamines and nitrosamides) [1]. An Acceptable Daily Intake (ADI) of nitrate, 3.7 mg/kg body weight/day (equivalent to 222 mg nitrate per day for a 60 kg adult), was established by the Food and Agriculture Organization of the United Nations and the World Health Organization (FAO/WHO)–Expert Committee on Food Additives (JECFA) in 2002 [1].

The most used methods for nitrate detection are colorimetric (after reduction to nitrite), UV detection, ion-exchange chromatography and potentiometry with ion-selective electrodes [4,5,6]. Amperometric methods represent an alternative for nitrate detection, however they require the use of suitable electrocatalysts, which can be both molecular or biomolecular catalysts [7,8,9], as well as special electrode materials [10,11,12], including copper and its alloys [13,14]. Voltammetric and amperometric methods of analysis furnish high sensitivity with a short response time, requiring cheap instrumentation and being suitable for direct or on-site analyses, even in colored samples.

In the frame of amperometric sensors, nanosized materials, that is, a wide family of materials with different shape and composition, present several advantages [15]. The synthesis of nano-objects under controlled conditions allows the formation of nanostructures directly onto the electrode surface, which reflects in an enhanced surface/volume ratio [15]. This plays an important role in electrocatalysis and in decreasing the overpotential involved in charge transfer processes. 

Membrane template assisted procedure (TAP), which consists of the use of a nanoporous membrane as a template to create a 2D nanostructured electrode surface, was proposed [16]. Such electrodes present a high S/N ratio, due to the characteristic geometric area and active area, that improves the analytical performance of the electrodes [17,18]. Such TAP can be applied in a different way to obtain an ensemble of nanowires, with a very high active area suitable as an electrocatalytic surface [19]. A crucial step in TAP is the removal of the template after nanostructuring. The use of O_2_ plasma for the controlled removal of the polycarbonate (PC) membrane in TAP for 3D-NEEs has been proposed [17,20]. This treatment is not suitable for some materials due to the oxidation effect of the electrode surface. To avoid unpleasant effects of O_2_ plasma, a chemical etching method based on the partial dissolution of the PC membrane in suitable solvent mixtures has also been proposed [20,21,22].

On the other hand, a low-temperature atmospheric plasma for surface cleaning is an interesting method due to its less aggressive effect on treated surfaces. Nowadays, it is used in a wide range of applications ranging from biomedical to cultural heritage interest. While in the first case it is applied mainly for sterilization and functional coating deposition purposes [23,24,25], in the latter, it is used for the cleaning of historical stone materials, ancient manuscripts, metallic surfaces, and others [25]. It also can be used in a reducing atmosphere to prevent formation or remove surface metal oxides.

In a previous study, our group developed an electrochemical template-assisted procedure to produce a copper-nanowires electrode ensemble (CuWNEE) for nitrate detection. Copper nanowires are finding increasing applications in a variety of fields, from electrical to optical [26]. For sensors development, we used electrodeposition of copper in track-etched polycarbonate (PC) membranes [27,19]. Chemical etching has been performed for template removal by repeatedly soaking the nanostructured electrode in dichloromethane (CH_2_Cl_2_). In the present study, we use a low-temperature atmospheric plasma to improve the etching procedure to better remove the PC template, obtaining a more active nanostructured surface suitable for nitrate determination in food samples and natural water. To this aim we propose to combine two etching treatments, i.e., a chemical etching followed by a low-temperature atmospheric plasma under reduction conditions, in order to prepare CuWNEEs with improved electrocatalytic properties, suitable for water and edible vegetables.

## 2. Materials and Methods

### 2.1. Materials

All chemicals were of analytical grade and used without further purification. Solutions were prepared with doubly distilled water (18.2 MΩ cm). Reagents used were Nafion® 117 at 5% w/v solution; H_2_SO_4_; NaCO_3_ and NaHCO_3_ (Sigma–Aldrich, Merck KGaA, Darmstadt, Germany); CuSO_4_ (Carlo Erba Regents, Milan, Italy); Na_2_SO_4_ (Fluka, Buchs, Switzerland); NaNO_3_ (Alfa Aesar, Karlsruhe, Germany).

### 2.2. Electrode Preparation

A potentiostatic deposition, as described by Stortini et al. [19], was used to grow copper nanowires into the pores of a PC membrane (400 nm pore diameter, thickness 10 μm, pore density 1 × 10^8^ pores cm^−2^, from SPI-pore™ (West Chester, PA, USA) following a template-assisted procedure. Briefly, the PC template was attached to the Cu-disk electrode by a thin layer of Nafion solution diluted to 0.5% with methanol. The PC membrane in contact with the Cu disk was pre-sputtered with a thin gold layer, with a calculated thickness of 75 nm, using a sputter coater (Cressington 108 auto, Watford, UK; target distance = 50 mm, i = 40 mA, t = 30 s in Ar atmosphere). Cu deposition was carried out at −0.250 V Ag/AgCl, KCl and sat for 120 s at room temperature in 0.6 M CuSO_4_, 10^−2^ M H_2_SO_4_ solution. Following deposition of the copper nanowires, the PC membrane was chemically dissolved by surface contact in CH_2_Cl_2_ for 60 s.

### 2.3. Plasma Treatment

After chemical etching, a plasma treatment with a low-temperature atmospheric plasma (Nadir S.r.l., Venice, Italy) [28] was applied to the nanostructured electrode. The plasma jet from a mini-torch is low power and low temperature and is obtained by a Dielectric Barrier Discharge configuration non - Local Thermodinamic Equilibrium (DBD non-LTE) [28]. The optimized working conditions of plasma were Ar 10 L min^−1^ 0.5% H_2_; distance from the torch = 2 mm; power high-frequency = 16 W; power radio-frequency = 30 W, time application = 60 s. The nanostructured electrodes obtained were kept protected from the oxygenated atmosphere in a proper container for use the same day. If the electrodes were not used on the same day, a fast activation could be done by plasma treatment, since the device was portable and user-friendly.

In brief, a low-temperature plasma set-up comprises a first pair of electrodes, singularly separated by a dielectric layer and externally positioned with respect to a tubular duct where the gas flows, and a second pair of electrodes, also singularly separated by a dielectric layer and externally positioned with respect to a tubular duct where the gas flows downstream. A high-frequency excitation is applied to the first pair of electrodes, while a radio-frequency excitation is applied to the second one. The plasma generated in this way emerges from the gas flow at the outlet of the transport duct. The high-voltage excitation can be applied in pulse trains and the radio-frequency generator is substantially activated for the purpose of limiting the thermal load on the treated substrate, allowing it, in proper conditions, to not exceed the temperature of 40 °C. Chemical precursors and reagents can be added to the plasma as vapors or aerosols by means of a coaxial central transport duct with the tubular duct for the gas, which facilitates treatments on the surface to be easily oxidizable or reducible. For instance, addition to the plasma of small percentages of hydrogen gas can allow it to operate in a chemically reducing environment [25]. The Plasma Jet device for the activation of copper nanowires was provided by Nadir S.r.l., Italy. More details and practical information, including schemes and figures of the use of the device, are available in the references cited above and others presented herein.

### 2.4. Electrochemical Measurements

Electrochemical measurements were performed at room temperature (23 ± 1 °C) under a nitrogen atmosphere with a CuWNEE [19] as a working electrode, in a conventional single-compartment cell equipped with a platinum wire counter electrode and a KCl-saturated Ag/AgCl reference electrode to which all potentials reported here are referred. For electrochemical measurements, a CHI1000 workstation (CH Instruments, Austin, TX, USA) controlled via PC by its own software was used. Voltammetric measurements were drawn from linear sweep voltammetry (LSV), from −0.2 to −0.8 V, at a scan rate of 10 mV s^−1^. A previous application of a cathodic potential would minimize the oxides, and activate the electrode surface. Several LSVs were recorded at different CuWNEEs in pure supporting electrolyte. This was to obtain blank voltammograms for subtraction in the nitrate determination using standard addition methods in all the samples.

### 2.5. Samples Preparation

Samples of water from the river Zero, located in the Venice province, were collected with a clean bottle and immediately filtered with a polycarbonate membrane, 0.45 µm pore diameter, and analyzed within 24 h.

Leafy vegetables analyzed were chard and rocket salad or rucola. The European Standard method for determination of nitrate and/or nitrite content BS EN 12014-4:2005 was used for the extraction of nitrate from vegetables [29]. In short, 10 g of chopped sample were submerged in water at a temperature of 50–60 °C, mixed thoroughly with a homogenizer and then transferred to a 200 mL volumetric flask and filled with water to the nominal volume. Afterwards, the extract was filtered with a polytetrafluoroethylene (PTFE) 0.45 μm pore size membrane for aqueous solutions. If the solution obtained was clear, no further filtration was necessary before introducing the sample in the electrochemical cell. Otherwise, a further filtration was performed before taking measurements. Assessments of NO_3_^−^ in food and water were performed within 24 h after sample extraction or sample filtration.

Natural water samples and vegetable extracts were adjusted at pH 3.0 with 0.1 M H_2_SO_4_ before voltammetric measurements. Eventual dilution with 0.1 M H_2_SO_4_ was performed when required.

### 2.6. Ion Chromatography (IC)

Metrohm 761 IC chromatograph equipped with a polyvinyl alcohol column with quaternary ammonium groups (IC Anion Column Phenomenex STAR ION A300, Metrohm, Herisau, Switzerland) was used for chromatographic analysis. After chemical suppression, 1.8 mM sodium carbonate and 1.7 mM sodium hydrogen carbonate solution with a conductivity of 14 μS cm^−1^ were used as eluent.

### 2.7. Scanning Electron Microscopy

The scanning electron microscopy (SEM) observations were performed with a TM3000 Hitachi (Tokyo, Japan) tabletop scanning electron microscope.

## 3. Results and Discussion

### 3.1. Preparation and Characterization of Nanowires Array

**Figure 1:**
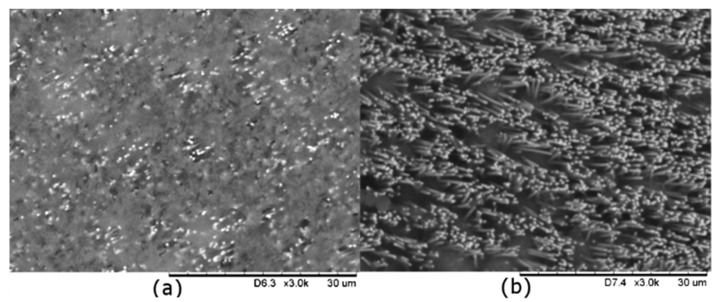
Scanning electron microscopy (SEM) images of CuWNEEs obtained by (**a**) template deposition after chemical etching; and (**b**) after low temperature plasma etching of CuWNEE reported in (**a**).

At first, after template deposition, a single step chemical etching was carried out, putting in contact the CuWNEEs with CH_2_Cl_2_ for 60 s. The SEM image in Figure 1A shows that the CuWNEEs (bright parts of the image) are still covered by a "cloud" of polycarbonate (foggy areas), providing evidence that just part of the template was removed [19].

In order to quickly obtain better cleaning of the nanostructures, a study of the effect of the use of low-temperature atmospheric plasma was completed operating under reducing conditions. The experimental parameters varied were the power high-voltage and radio-frequency, and the time of the treatment. As indicated in Table 1, it was observed that for treatment times higher than 60 s with power high voltage generator in Watt (HV) and power of radiofrequency generator in Watt (RF), spark episodes occurred on the nanowires. Therefore, 60 s, RF 30 and HV 16 were chosen as the optimized values from trial.

Figure 1B presents the SEM image of the same array as in Figure 1A, after the optimized treatment with low-temperature atmospheric plasma. The comparison of these images shows that the combination of these two treatments allows the almost complete removal of the PC template. The surface area of CuWNEE is cleaned up efficiently by the soft plasma etching used here. The final results obtained are comparable with those obtained on arrays of gold nanowires using plasma etching under more aggressive conditions of oxygen plasma [22].

**Figure 2:**
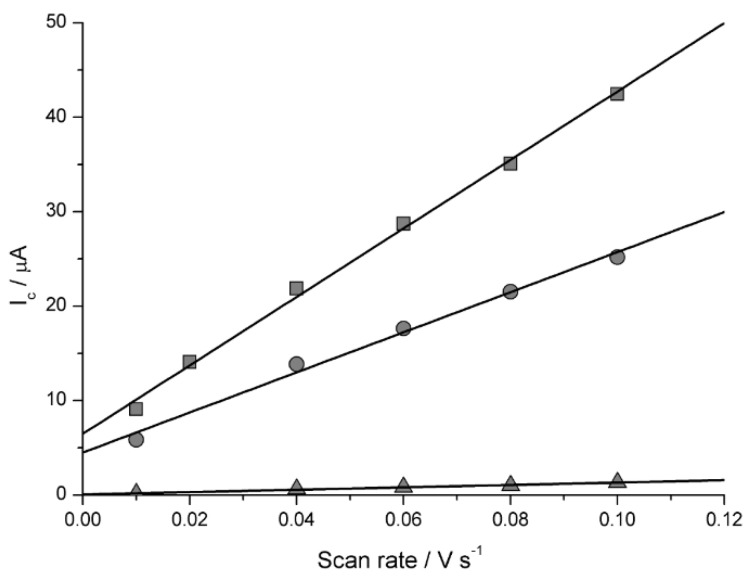
Capacitive current (*I*_c_) vs scan rate for nanostructured copper electrodes (CuWNEE) after chemical and low-temperature plasma etching (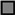), after only chemical etching (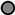) and flat (CuE) copper electrode (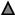).

The determination of *A*_act_ of the CuWNEEs after chemical and plasma treatment was carried out by cyclic voltammetry (CV) in pure supporting electrolyte (0.1 M Na_2_SO_4_) at different scan rates from 0.01 to 0.1 V s^−1^. Figure 2 presents the values of the capacitive current (*I*_c_) as a function of the scan rate (*v*) obtained from CVs for CuWNEEs, measured as the half-difference between the anodic and cathodic current measured at −0.3 V [30,31,32]. From this figure, it is evident that the linear dependence of *I*_c_ on the scan rate, which is in agreement with the theory and given by the equation:

*I*_c_ = *C*_dl_*A*_act_*v* (1)

where *C*_dl_ is the double layer capacitance [30], *A*_act_ is the active area and *v* is the scan rate. From the slope of the linear equation (362 μA V^−1^ s) and considering *C*_dl_ for copper as equal to 166 μF s^−1^ [31,32], an *A*_act_ of 2.06 cm^2^ was calculated. The comparison of this value with that obtained with only chemical etching (namely, 1.26 cm^2^) indicates that plasma treatment increased by a further 64% at the available active area.

### 3.2. Determination of NO_3_^−^ in Real Samples

#### 3.2.1. River Water

**Figure 3:**
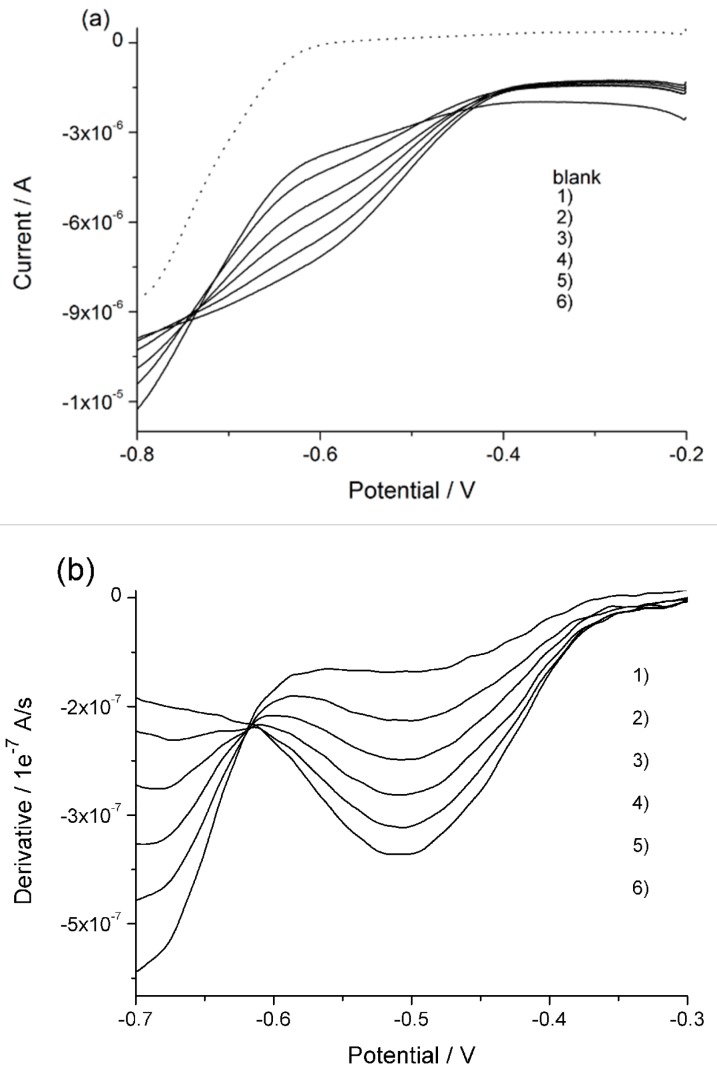

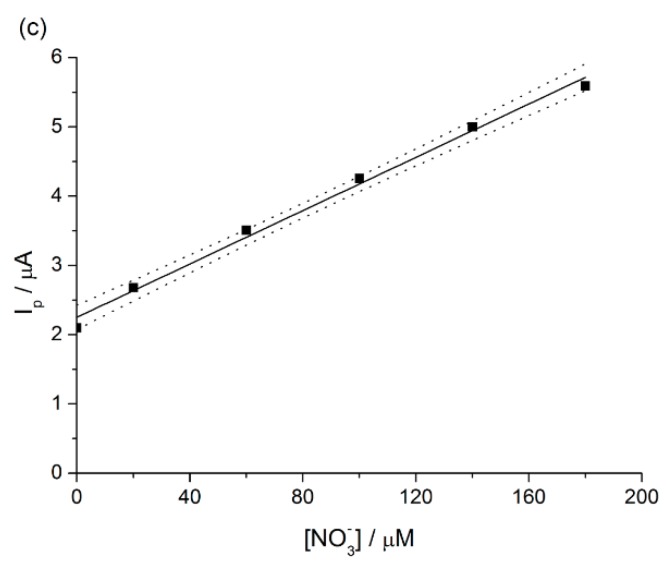
(**a**) Linear sweep voltammograms recorded in acidified river water at 10 mV s^−1^ (blank: Dotted line); (**b**) first derivative of LSVs reported in (**a**); (**c**) calibration plot, with confidence bands at 90%, obtained by standard additions for the following concentrations in μM: 1) 0, 2) 20, 3) 60, 4) 100, 5) 140, 6) 180.

Figure 3a presents the linear sweep voltammograms with the CuWNEE treated as described in the previous section, recorded in the filtered river water at pH 3 before and after NaNO_3_ standard additions. The blank, that is, the voltammogram recorded in only supporting electrolyte, is plotted as well (dotted line in Figure 3a). These voltammograms are characterized by rather broad reduction peaks with a peak current which indicates a concentration of nitrate in the sample in the order of a few hundred micromolar. For this reason, the standard addition of nitrate was in the range of 20–40 µM. At such a low concentration level, even with nanostructured copper electrodes, the reduction signals are not fully distinguishable from the background. Moreover, it can be noted that the nitrate reduction is not detected at a fixed potential, but can fluctuate within a potential range, typically –0.7 and –0.5 V [32], depending on the state of the surface of the electrode and the supporting electrolyte. In order to determine the correct peak potential value to perform quantitative analyses, the first derivative was calculated for each voltammogram. The relevant plots are presented in Figure 3b. The point where the derivative is constant is assumed to be the peak potential and it is located at –0.620 V. This value is < –0.7 V, observed on CuWNEE and treated by only chemical etching [19], indicating an improved electrocatalytic activity for plasma-cleaned CuWNEEs. From the standard addition plot shown in Figure 3c, a nitrate concentration of 117 µM (7.3 mg L^−1^) in the sample was measured.

It can be noted that the LSVs in Figure 3a are characterized by an isopotential point at −0.75 V. The presence of this singular point derives from the evidence that for E > −0.75 V, the current increases with increasing nitrate concentration (producing the above described nitrate reduction peak) while for E < −0.75, the current scale inverts with the nitrate concentration. A similar behavior was previously observed when nitrate was analyzed with CuWNEE in solutions containing chloride anions [19]. In the experimental conditions used in this work, we cannot discount the leakage of some chloride from the Ag/AgCl, KCl reference electrode. However, the possible role related to the formation of copper oxides on the surface of the copper nanowires at the beginning of the scan cannot be ignored [26]. Detailed studies of this issue are in progress.

#### 3.2.2. Vegetable Samples

**Figure 4:**
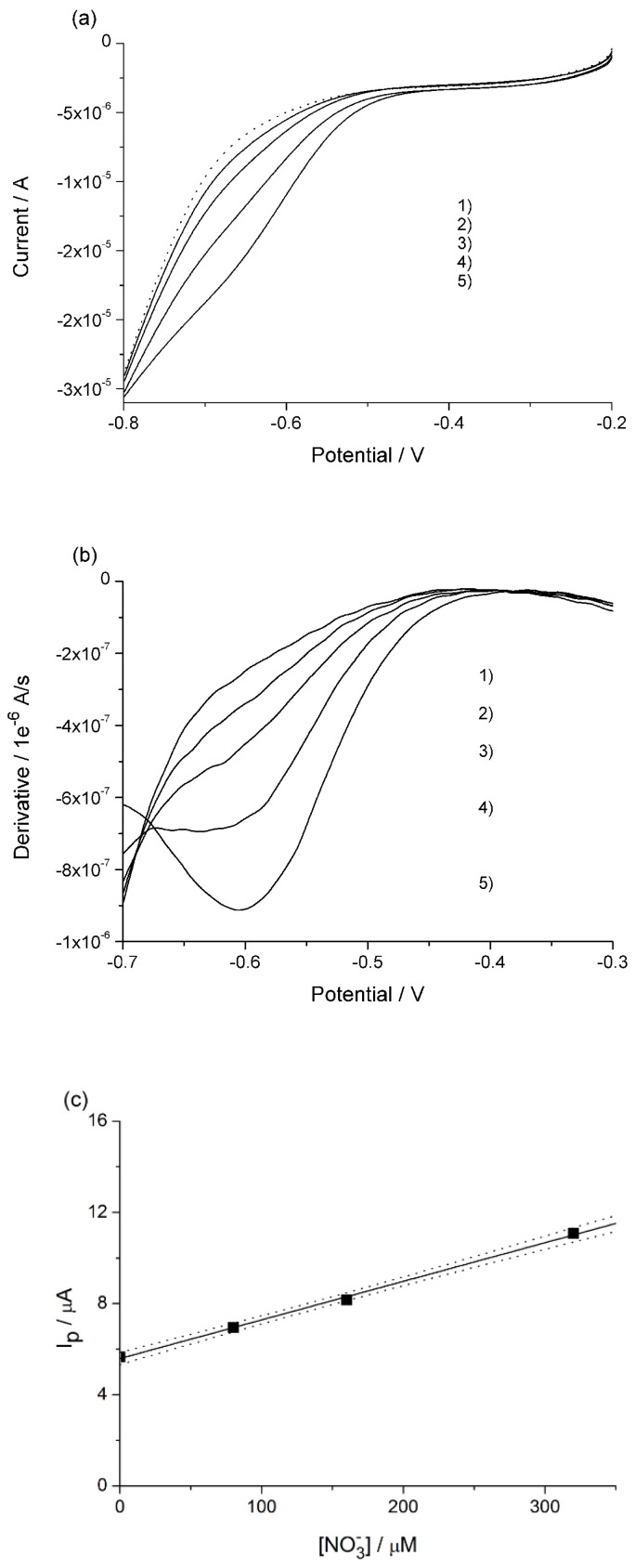
(**a**) Linear sweep voltammograms recorded in 10-times diluted rucola extract acidified, at scan rate of 10 mV s^−1^; (**b**) first derivative of LSV reported in (**a**); (**c**) calibration plot, with confidence bands at 90%, obtained by standard additions of nitrate of the following concentrations in μM: 1) 0, 2) 80, 3) 160, 4) 320, 5) 640.

**Figure 5:**
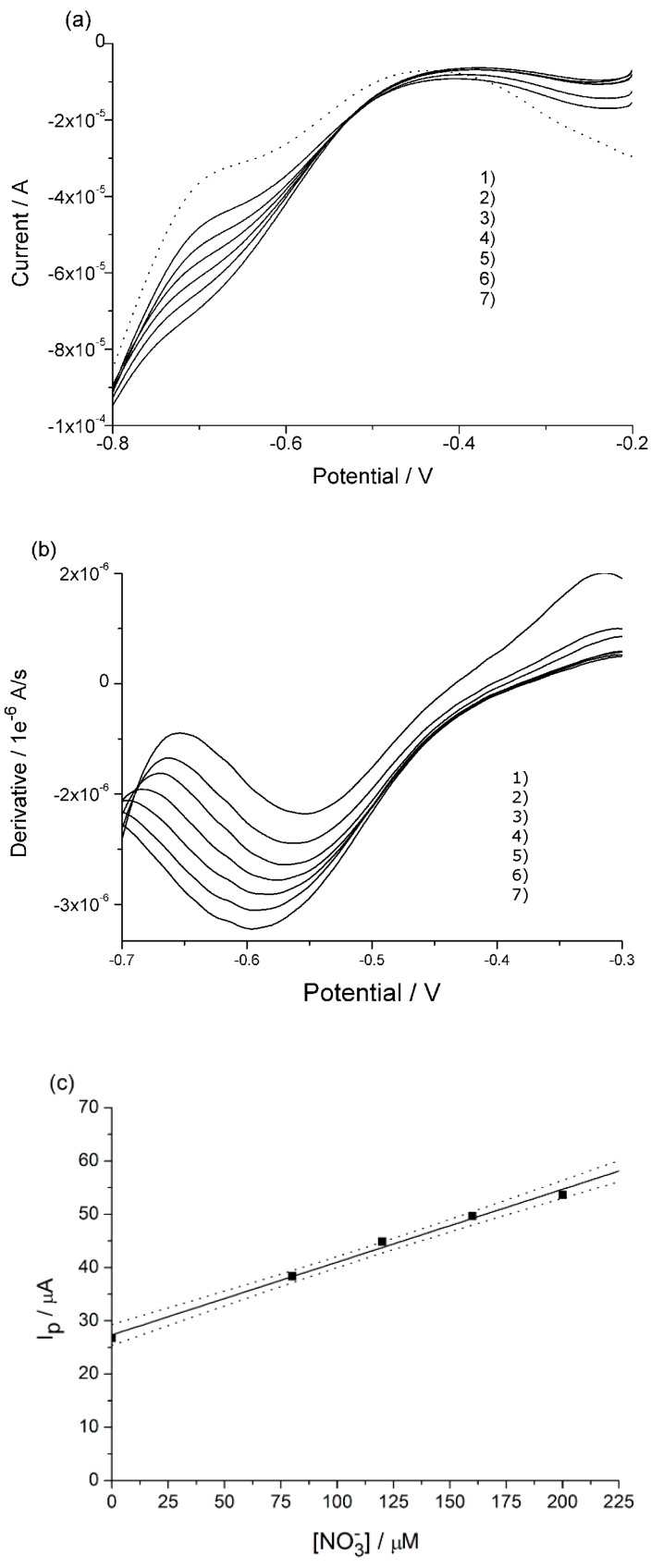
(**a**) Linear sweep voltammograms recorded in 5-times diluted chard extract acidified, at scan rate of 10 mV s^−1^; (**b**) first derivative of LSV reported in (**a**); (**c**) calibration plot, with confidence bands at 90%, obtained by standard additions of nitrate in the following concentrations in μM: 1) 0, 2) 80, 3) 120, 4) 160, 5) 200, 6) 240, 7).

The extraction procedure of nitrate described in the Materials and Methods was applied to edible leafy vegetables, namely rocket salad (rucola) and chard. On the basis of preliminary tests, the extracts were diluted 10 times and 5 times, respectively, with 0.1 M H_2_SO_4_. This dilution was performed both to lower the high NO_3_^−^ concentration and to minimize the possible effect of dissolved organic matter. LSVs of the sample before and after the nitrate standard additions were recorded and are shown in Figure 4a and Figure 5a. They presented similar patterns as those obtained from the water samples, so the same procedure for calculating the first derivative for river water as described above (see Figure 4b and Figure 5b) was applied. Figure 4c and Figure 5c report the respective standard addition plots where currents, after blank subtraction, were measured at −0.685 V and −0.695 V for rucola and chard, respectively. The concentrations of nitrate measured in the electrochemical cell were 4097 μM (205 ppm) and 999 μM (62 ppm), respectively. Concentration in the raw vegetables was calculated on the basis of the mass of vegetable analyzed. Relevant values are reported in the second column of Table 2.

Nitrate concentrations in the real samples have been validated by IC and relevant values are reported in the third column of Table 2. The comparison with the electrochemical data obtained by LSV with CuWNEEs indicates a satisfactory agreement.

For the river water sample in particular, the determined nitrate concentration was lower than the maximum concentration of 50 mg L^−1^, indicated by the European Directives (1991/676/EC, 1998/83/EC and 2003/40/EC) and the World Health Organization (WHO) in the guidelines for drinking-water quality [33]; and lower than the limit of 45 mg L^−1^, fixed by Italian regulations (Legislative Decree 2001/02/02/ n.31).

As far as edible leafy vegetables analysis is concerned, the values determined in this work are of the same order of magnitude as those of a previous study, where nitrate concentrations, determined by High Performance Liquid Chromatography (HPLC) in chard samples produced in different seasons in Zagreb, were 1049.40 and 2260.20 mg kg^−1^ [2]. Moreover, in Swiss chard species grown with organic fertilizers, nitrate levels determined by a modified cadmium–Griess method were much higher, averaging across the different samples and species at 2778.6 ± 1474.7 mg kg^−1^ [33,34]. Rucola is considered one of the leafy vegetables with the most consistent amount of nitrate and according to reports by the Scientific Opinion of the Panel on Contaminants in the Food Chain, the consumption of more than 47 g of rucola at the median nitrate concentration of 4800 mg kg^−1^ would exceed the ADI without taking into account any other vegetable [1]. In our case, values obtained with both techniques are lower than this median value.

## 4. Conclusions

Nanostructured electrodes can easily be obtained by an electrochemical template-based procedure (TAP). In this work, a nanoporous polycarbonate membrane was used as a template for the preparation of CuWNEEs. The membrane etching is a very important step to removing the template and achieving the maximum active area of the electrode. The application of a chemical etching step combined with a low-temperature plasma treatment allowed us to improve the active area and the performance of the CuWNEEs in the determination of nitrate. The electrodes were applied to the electrochemical determination of nitrate in river water and leafy vegetables, and the results were in agreement with ion chromatographic and literature data.

## Figures and Tables

**Table 1:** Low-temperature atmospheric plasma trials on CuWNEE. Gas flow Ar 10 L min^−1^ 0.5% H_2_ and distance from the torch 2 mm.

Electrode	Test	Power HV (W)	Power RF (W)	Time (s)	Spark Event
E3	1°	16	30	60	–
E3	2°	16	35	90(+30)	+
E3	3°	16	30	180(+90)	+
E2	1°	16	30	180	+
E1	1°	16	30	360	+
E5	1°	70	30	60	+

**Table 2:** Nitrate concentration values determined by electrochemical measurement (LSV) and ion chromatography (IC).

Sample	Electrochemical	Ion Chromatography	Relative Variation %
River water	7.3 ± 0.5 mg L^−1^	7.5 ± 0.4 mg L^−1^	3.3
Rucola	4097 ± 0.6 mg kg^−1^	5016 ± 0.4 mg kg^−1^	8.3
Chard	1239 ± 0.7 mg kg^−1^	1352 ± 0.4 mg kg^−1^	18.3
